# Atractylenolide II Suppresses Glycolysis and Induces Apoptosis by Blocking the PADI3-ERK Signaling Pathway in Endometrial Cancer Cells

**DOI:** 10.3390/molecules29050939

**Published:** 2024-02-21

**Authors:** Shuang Tian, Lili Ren, Chao Liu, Zhe Wang

**Affiliations:** 1 Department of Pathology, College of Integrated Chinese and Western Medical, Liaoning University of Traditional Chinese Medicine, Shenyang 110847, China; tianshuang@jzmu.edu.cn; 2 Department of Cell Biology and Genetics, Basic Medical College, Jinzhou Medical University, Jinzhou 121001, China; 3 Department of Neurobiology, Basic Medical College, Jinzhou Medical University, Jinzhou 121001, China; jyrenlili@jzmu.edu.cn

**Keywords:** Atractylenolide II, PADI3, glycolysis, apoptosis, endometrial cancer, ERK

## Abstract

Atractylenolide II (AT-II), the major bioactive compound of *Atractylodes macrocephala*, exhibits anti-cancer activity against many types of tumors, but the roles and the potential mechanisms in endometrial cancer remain unclear. In the present study, AT-II treatment was found to significantly suppress RL95-2 and AN3CA cell proliferation and glycolysis, and induced their apoptosis by inactivating the ERK signaling pathway, accompanied by the changing expression of the glycolytic key enzymes and apoptotic-related proteins. Peptidyl arginine deiminase 3 (PADI3), as the candidate target gene of AT-II, was highly expressed in the endometrial cancer tissues and associated with a poor prognosis according to bioinformatics analysis. PADI3 knockdown inhibited proliferation and glycolysis in endometrial cancer cells and induced cell apoptosis. Furthermore, AT-II negatively regulated the expression of PADI3, and PADI3 overexpression reversed the effects of AT-II on endometrial cancer cells. Our findings suggested that the anti-cancer function of AT-II is associated with the suppression of glycolysis and induction of apoptosis by blocking the PADI3-ERK signaling pathway. Thus, AT-II represents a novel therapeutic target for endometrial cancer and targeting AT-II may serve as a potential strategy for the clinical therapy of endometrial cancer.

## 1. Introduction

Endometrial cancer is the sixth most common cancer in women with high incidence and mortality rates [1]. Recent epidemiological investigation data have revealed that there were approximately 41.7 million new cases of and 9.7 million deaths due to endometrial cancer in 2020 worldwide [2]. In recent years, the incidence rate of endometrial cancer in China has also increased year by year, especially in Beijing and Shanghai, where endometrial cancer has ranked first with regard to the incidence rate of malignant tumors in women instead of breast and ovarian cancers [3]. The most common therapeutic method for endometrial cancer is still surgical resection, assisted with chemotherapy and radiotherapy. However, long-term therapy puts tremendous physical and psychological pressure on patients [4]. Therefore, it is urgent to find a novel agent that can improve the cure rate and the life quality of patients with endometrial cancer.

**Figure 1:**
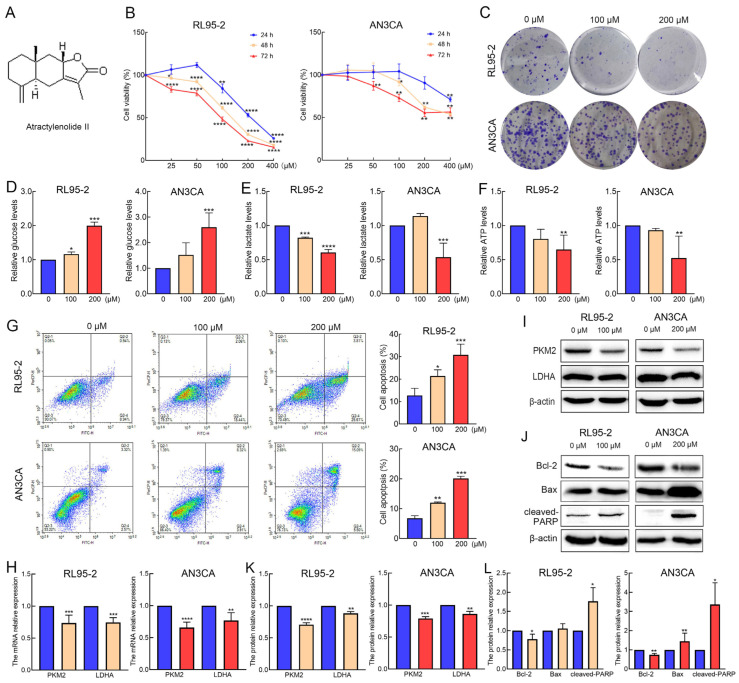
AT-II suppresses the proliferation and glycolysis in endometrial cancer cells and induces their apoptosis. (**A**) The chemical structure of AT-II. (**B**) RL95-2 (left panel) and AN3CA (right panel) cells were treated with AT-II at different concentrations for 24 h, 48 h, and 72 h. Cell viability was examined by CCK-8 assay. (**C**) AT-II reduced the number of colonies in a dose-dependent manner. (**D**) AT-II suppressed the consumption of glucose leading to the increasing level of glucose in RL95-2 and AN3CA cells. (**E**,**F**) AT-II decreased the production of lactate (**E**) and ATP (**F**). (**G**) Cell apoptosis rates were tested by flow cytometry. (**H**) The mRNA expression levels of PKM2 and LDHA were decreased in response to AT-II treatment. (**I**) The protein expression levels of PKM2 and LDHA were decreased after AT-II treatment. (**J**) The expression of Bcl-2, Bax and cleaved-PARP were detected by Western blot after AT-II treatment. (**K**) The protein-relative expression levels of PKM2 and LDHA were quantified by Clinx Gel Analysis software (Version 2.6.1.0). (**L**) The protein-relative expression levels of Bcl-2, Bax and cleaved-PARP were quantified by Clinx Gel Analysis software. All experiments were replicated independently at least three times and presented as mean ± SD. * *p* < 0.05, ** *p* < 0.01, *** *p* < 0.001, **** *p* < 0.0001 vs. 0 µM group.

*Atractylodes macrocephala* (known as *baizhu* in China) is a common plant usually used in combination with other traditional Chinese medicines for the clinical treatment of cancers [5]. Atractylenolide II (AT-II, the chemical structure is shown in Figure 1A) is the major bioactive compound isolated from the Rhizome of *Atractylodes macrocephala*, which shows a wide range of pharmacological effects, especially anti-cancer activity [6]. Previous studies have reported that AT-II inhibits cell growth and metastasis, arrests the cell cycle, induces apoptosis, and reverses chemo-resistance in multiple types of tumors by regulating various molecular pathways [7,8,9,10,11,12]. However, the effects of AT-II on human endometrial cancer and the potential mechanisms underlying these effects remain elusive.

One of the hallmarks of cancer cells is the altered energy metabolism model [13]. Cancer cells commonly prefer to use glucose via the glycolytic pathway to produce energy even under adequate oxygen conditions, which is known as the Warburg effect [14]. This model of glucose metabolism reprogramming results in a changed microenvironment, increased ATP levels, and enhanced drug resistance, thereby contributing to the escape of cancer cells from apoptosis and the enhancement of their growth. Therefore, the disruption of glycolytic progression by inhibiting the activity of key glycolytic regulatory enzymes, including pyruvate kinase M2 (PKM2) [15] and lactate dehydrogenase A (LDHA) [16], has become a novel direction in the development of anti-cancer drugs. Peptidyl arginine deiminase 3 (PADI3) belongs to the peptidyl arginine deiminase family and is a post-translational protein modification enzyme that can catalyze the conversion of arginine to citrulline, and this progress is known as citrullination. It has been reported that PADI3 is highly expressed in some solid tumors, such as bladder, pancreatic, cervical, head and neck, and clear-cell renal cancers, in which increased PADI3 enhances the citrullination of the glycolytic rate-limiting enzyme PKM2, leading to increased levels of glycolysis [17,18]. Nevertheless, the roles of PADI3 in endometrial cancer have not been previously explored.

In our study, we found that AT-II inhibited glycolysis and induced cell apoptosis by blocking the PADI3-ERK signaling pathway, thereby suppressing endometrial cancer cell growth. These findings provide valid data and highlight AT-II as a new target for future clinical applications in the treatment of patients with endometrial cancer.

## 2. Results

### 2.1. AT-II Suppresses Proliferation and Glycolysis in Endometrial Cancer Cells and Induces Their Apoptosis

The RL95-2 and AN3CA cells were treated with 0, 25, 50, 100, 200, and 400 µM of AT-II and tested using the CCK-8 assay. The results showed that AT-II significantly inhibited cell viability in a time- and dose-dependent manner (Figure 1B). Moreover, RL95-2 cells seemed more sensitive to AT-II than AN3CA cells. When RL95-2 cells were exposed to 100 µM of AT-II for 48 h, the cell viability was reduced to nearly 50%, while AN3CA cells needed 200 µM of AT-II treatment. Therefore, we selected 100 µM and 200 µM as appropriate concentrations of AT-II for the following experiments. The colony formation capability assays revealed similar results, and AT-II reduced the number of colonies in a dose-dependent manner (Figure 1C and Figure S1). AT-II treatment also suppressed glycolysis by decreasing the consumption of glucose and the production of lactate and ATP (Figure 1D–F), accompanied by decreased the mRNA and protein expression levels of PKM2 and LDHA (Figure 1H,I,K). Furthermore, AT-II induced apoptosis in RL95-2 and AN3CA cells in a dose-dependent manner (Figure 1G). The decreased expression of B-cell lymphoma-2 (Bcl-2) and the increased expression of cleaved-poly ADP-ribose polymerase (cleaved-PARP) were detected by Western blot in both two cells. The protein expression level of Bax was elevated only in AN3CA cells (Figure 1J,L).

### 2.2. PADI3 Shows Good Prognostic Value and May Serve as a Novel Clinical Indicator for Endometrial Cancer

**Figure 2:**
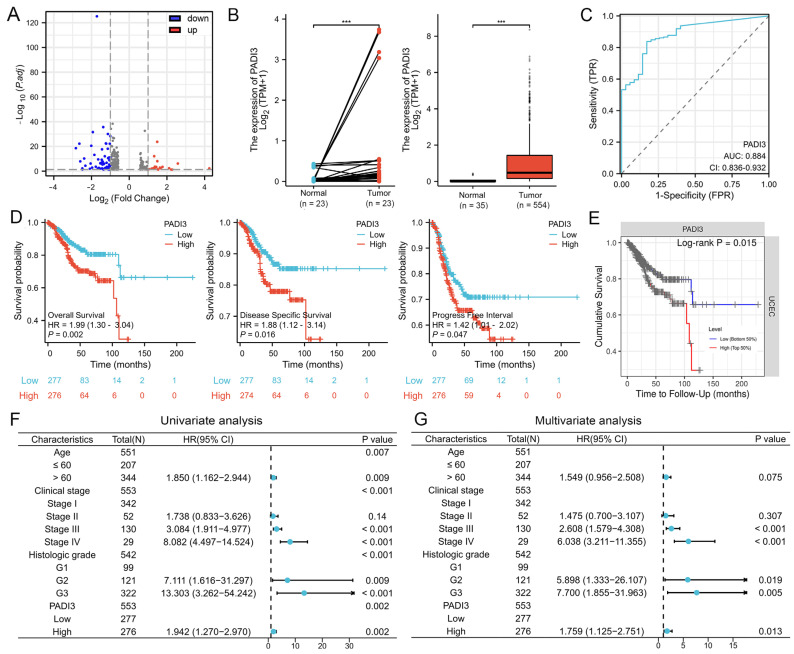
PADI3 is upregulated in patients with endometrial cancer and correlates with poor prognosis. (**A**) Volcano plot showed a total of 82 DEGs between the AT-II treatment group vs. DMSO treatment group by RNA sequencing and the mRNA of PADI3 was downregulated in response to AT-II treatment in RL95-2 cells. (**B**) PADI3 was highly expressed in endometrial cancer between paired (left panel) or non-paired (right panel) normal and endometrial cancer tissues (*** *p* < 0.001 vs. Normal tissues). (**C**) The ROC curve of PADI3 was detected. (**D**) The relationship between PADI3 and overall survival, disease-specific survival, and progress-free interval were verified. (**E**) The correlation between PADI3 and overall survival was analyzed using the TIMER database. (**F**,**G**) PADI3 was an independent risk factor for poor prognosis by the univariate (**F**) and multivariate (**G**) Cox regression analysis.

Transcriptome analysis was performed to elucidate the potential mechanisms underlying the effects of AT-II in endometrial cancer cells. The PADI3 mRNA expression was downregulated in response to AT-II treatment, as per the volcano plot analysis (Figure 2A). As a candidate target, we evaluated whether PADI3 played an important role in the progression of endometrial cancer and had meaningful clinical value. The data from the TCGA database were visualized using the Xiantao Academic online tool and the results showed that PADI3 mRNA was highly expressed in cancer tissues between paired or non-paired normal and endometrial cancer tissues (Figure 2B). The ROC curve suggested that PADI3 was a valuable diagnostic biomarker because of its high area under the curve value (Figure 2C). The expression level of PADI3 is positively related to the histologic grade, but not related to the age and clinical stage (Figure S2, Table S1). The expression level of PADI3 was negatively correlated with overall survival, disease-specific survival, and progress-free interval of patients with endometrial cancer. The expression level of PADI3 was higher, and the survival period was shorter (Figure 2D), consistent with the results from the TIMER database (Figure 2E). The univariate and multivariate Cox regression analysis data showed that PADI3 was an independent risk factor for poor prognosis (Figure 2F,G). These findings suggested that PADI3 had good prognostic value and played an important role in the progress of endometrial cancer. Thus, it may serve as a novel target for the treatment of endometrial cancer in clinical settings. 

### 2.3. PADI3 Was Negatively Regulated by AT-II in RL95-2 and AN3CA Cells 

**Figure 3:**
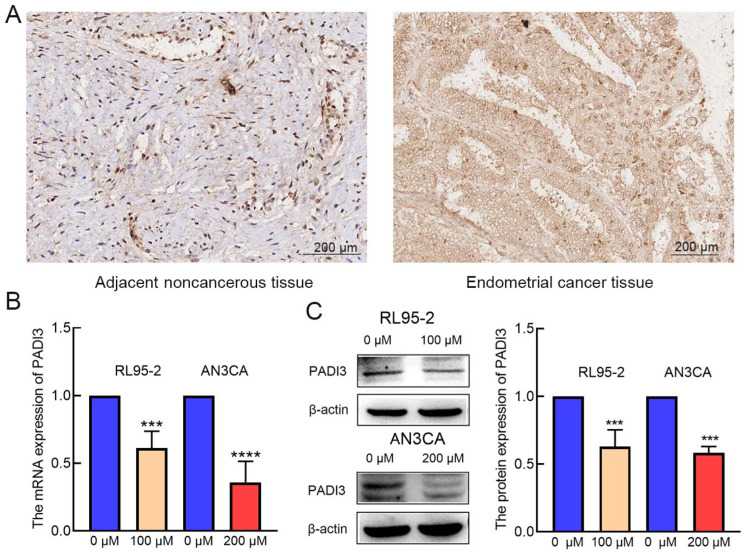
PADI3 was negatively regulated by AT-II. (**A**) The expression of PADI3 was confirmed by immunohistochemistry staining (Bar = 200 µm). (**B**) The mRNA of PADI3 was detected by real-time PCR PADI3 after AT-II treatment in RL95-2 and AN3CA cells. (**C**) The protein expression of PADI3 was tested by Western blot and quantified after AT-II treatment in RL95-2 and AN3CA cells. All experiments were replicated independently at least three times and presented as mean ± SD. *** *p* < 0.001, **** *p* < 0.0001 vs. 0 µM group.

To verify the above hypothesis, we detected the expression of PADI3 by immunohistochemistry staining using endometrial cancer tissue microarray. The results showed that PADI3 was highly expressed in endometrial cancer tissues (Figure 3A). Real-time PCR and Western blot analyses indicated that the mRNA and protein levels of PADI3 were downregulated after AT-II treatment (Figure 3B,C).

### 2.4. Knockdown of PADI3 Inhibits Proliferation and Glycolysis in RL95-2 and AN3CA Cells and Induces Their Apoptosis 

**Figure 4:**
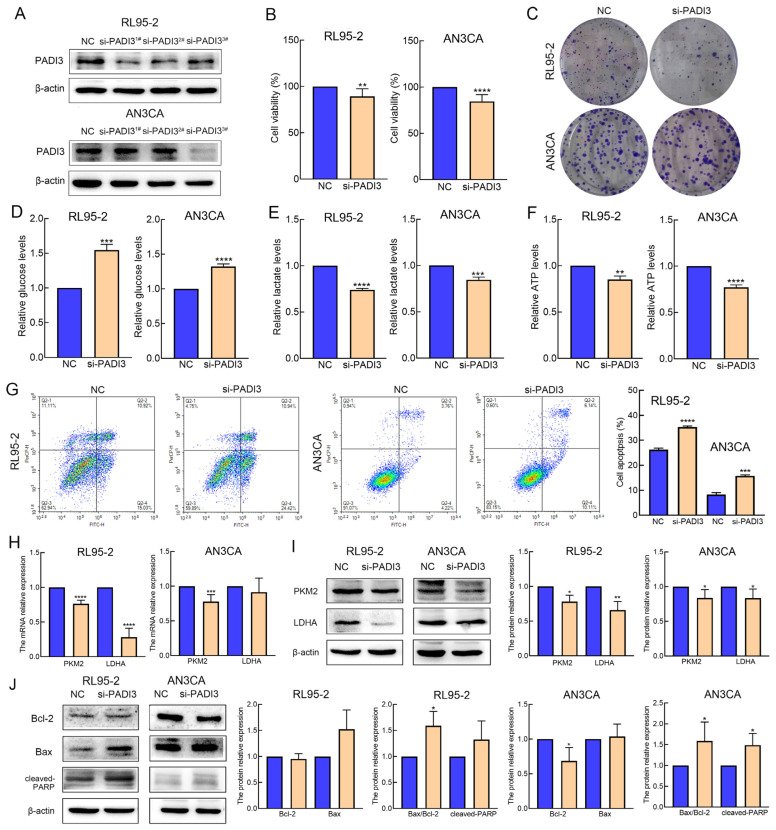
Depletion of PADI3 suppresses cell proliferation and glycolysis and induces their apoptosis in RL95-2 and AN3CA cells. (**A**) The expression of PADI3 was confirmed by Western blot analysis to validate the efficacy of PADI3 knockdown. (**B**) PADI3 negatively regulated cell viability. (**C**) PADI3 reduced the number of colonies in RL95-2 and AN3CA cells. (**D**–**F**) PADI3 knockdown inhibited cell glycolysis by decreasing the glucose consumption (**D**) and, lactate (**E**) and ATP (**F**) production. (**G**) PADI3 knockdown promotes the rates of cell apoptosis in RL95-2 and AN3CA cells. (**H**) PADI3 knockdown downregulated the mRNA expression of PKM2 and LDHA. (**I**) PADI3 knockdown downregulated the protein expression of PKM2 and LDHA. (**J**) PADI3 knockdown changed the expression levels of Bcl-2, Bax and cleaved-PARP. All experiments were replicated independently at least three times and presented as mean ± SD. * *p* < 0.05, ** *p* < 0.01, *** *p* < 0.001, **** *p* < 0.0001 vs. NC group.

PADI3 siRNA was transiently transfected into RL95-2 and AN3CA cells to identify the biological roles of PADI3 in endometrial cancer. First, Western blot was performed to validate the efficacy of PADI3 knockdown. The three PADI3 siRNAs were valid in RL95-2 cells and there was no significant difference in the inhibition efficiency among them, whereas only si-PADI3^3#^ was efficient in AN3CA cells (Figure 4A and Figure S3). Therefore, si-PADI3^3#^ was selected for use in the following experiment. Second, we assessed the cell viability, colony formation capability, glycolysis level, and apoptosis rate after transfecting the cells with si-PADI3 for 48 h. The data revealed that the RL95-2 and AN3CA cells from the si-PADI3 group exhibited lower cell viability, fewer colony numbers, lower consumption of glucose and production of lactate and ATP, and higher apoptosis rates, than those from the NC group (Figure 4B–G and Figure S4). Real-time PCR results indicated the mRNA expression of PKM2 and LDHA reduced after si-PADI3 treatment (Figure 4H). Western blot analysis showed that the expression of PKM2 and LDHA decreased significantly, and the ratio of Bax/Bcl-2 and the expression of cleaved-PARP increased after si-PADI3 treatment in RL95-2 and AN3CA cells (Figure 4I,J).

### 2.5. Overexpression of PADI3 Reverses the Roles of AT-II in RL95-2 and AN3CA Cells 

**Figure 5:**
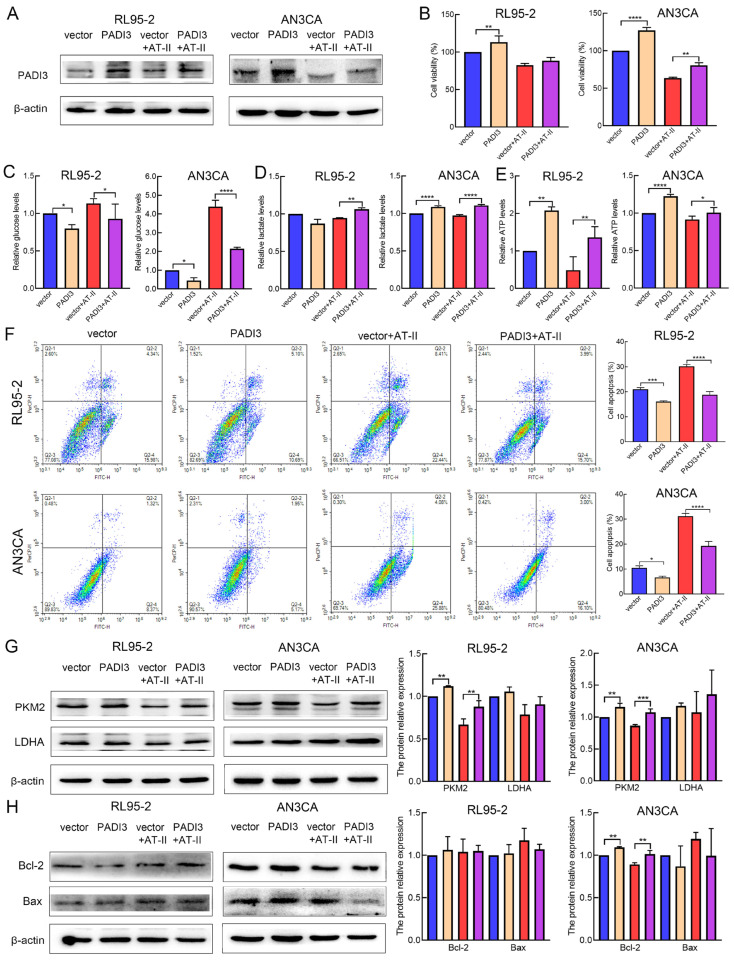
Overexpression of PADI3 attenuated the anti-cancer effects of AT-II. (**A**) The protein expression level of PADI3 was detected using Western blot after transfecting PADI3-overexpressing plasmid and treating with 100 µM or 200 µM AT-II for 24 h in RL95-2 and AN3CA cells, respectively. (**B**) PADI3 reversed AT-II-induced cell viability inhibition. (**C**–**E**) PADI3 attenuated AT-II-induced glycolysis suppression by promoting the consumption of glucose and the production of lactate and ATP. (**F**) PADI3 reduced AT-II-induced apoptosis. (**G**) The protein expression levels of PKM2 and LDHA were detected by Western blot analysis. (**H**) PADI3 overexpression attenuated the AT-II-induced decrease in the expression of Bcl-2. All experiments were replicated independently at least three times and presented as mean ± SD. * *p* < 0.05, ** *p* < 0.01, *** *p* < 0.001, **** *p* < 0.0001.

To further confirm PADI3 as the direct target of AT-II, we overexpressed PADI3 by transfecting RL95-2 and AN3CA cells with a PADI3-overexpressing plasmid. After transfection for 6 h, the cells were treated with DMSO or AT-II for another 24 h. Western blot analysis found that the expression level of PADI3 was remarkably increased in the PADI3 group compared with the vector group (Figure 5A and Figure S5). Moreover, we found that the PADI3 overexpression promoted cell ability, the consumption of glucose or the production of lactate and ATP, and reduced the apoptosis rates in RL95-2 and AN3CA cells (Figure 5B–F). Western blot analysis showed that the expression of PKM2 was significantly increased in RL95-2 and AN3CA cells (*p* < 0.01) (Figure 5G,H). These data confirmed that PADI3 served as an oncogene to promote the process of endometrial cancer, which was consistent with the results of PADI3 knockdown. In the rescue study, we found that PADI3 overexpression attenuated the AT-II-induced inhibition of cell viability and glycolysis, and promotion of cell apoptosis. Western blot results suggested that the AT-II-induced decrease in the expression of PKM2 was significantly reversed in both RL95-2 and AN3CA cells, and the expression of Bcl-2 was increased only in AN3CA cells when PADI3 was overexpressed (*p* < 0.01) (Figure 5G,H).

### 2.6. AT-II Inhibits Glycolysis and Induces Apoptosis in Human Endometrial Cancer Cells by Inactivating the ERK Signaling Pathway 

**Figure 6:**
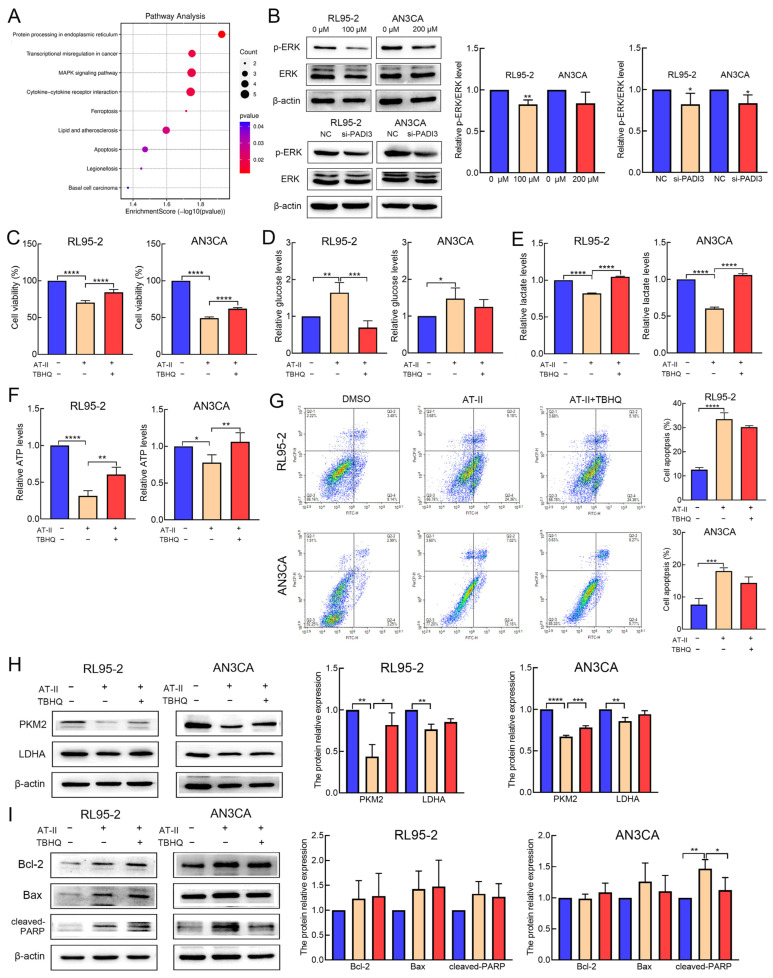
AT-II inhibited glycolysis and induced apoptosis by inactivating the ERK signaling pathway. (**A**) The results of KEGG enrichment show that genes enriched in the MAPK signaling pathway. (**B**) The expression of p-ERK1/2 and ERK were detected by Western blot. (**C**) RL95-2 cells were treated with DMSO, 100 µM AT-II, or 100 µM AT-II combination with 6.25 µM TBHQ for 24 h. AN3CA cells were treated with DMSO, 200 µM AT-II, or 200 µM AT-II combination with 12.5 µM TBHQ for 24 h. Cell viability was examined by CCK-8 assay and TBHQ-reversed AT-II-induced cell viability inhibition. (**D**) TBHQ attenuated AT-II-induced the consumption of glucose and the production of lactate (**E**) and ATP (**F**) suppression. (**G**) TBHQ reduced AT-II-induced apoptosis rates in RL95-2 and AN3CA cells. (**H**,**I**) The protein expression levels of PKM2, LDHA, Bcl-2, Bax, and cleaved-PARP were tested by Western blot. All experiments were replicated independently at least three times and presented as mean ± SD. * *p* < 0.05, ** *p* < 0.01, *** *p* < 0.001, **** *p* < 0.0001.

The mitogen-activated protein kinase (MAPK) signaling pathway is an important signaling network in cell proliferation, apoptosis, migration, and metabolism [19]. In our study, 82 DEGs were performed the analysis of Kyoto Encyclopedia of Genes and Genomes (KEGG) pathway enrichment. The results revealed that genes enriched in the MAPK signaling pathway (*p* = 0.018) (Figure 6A). Therefore, we examined the protein expression of phosphorylated-ERK (p-ERK) (Thr202/Tyr204) and total ERK. We found that AT-II treatment and PADI3 knockdown all reduced the protein expression of p-ERK1/2 (Figure 6B). To further explore whether ERK inactivation was involved in AT-II-induced glycolysis suppression and cell apoptosis promotion, the ERK activator tert-butylhydroquinone (TBHQ) was used. The RL95-2 and AN3CA cells treated with AT-II and TBHQ remarkably reversed AT-II-induced inhibition of cell viability and glycolysis, accompanied by the increased protein expression of PKM2, compared to the cases for the cells treated with AT-II alone (Figure 6C–F,H). Although the suppression of the AT-II-induced increase in the cell apoptosis rate by TBHQ was not marked (Figure 6G), the expression of cleaved PARP was increased in AN3CA cells (Figure 6I). These data indicated that ERK inactivation is essential for AT-II-induced glycolysis and cell apoptosis.

## 3. Discussion

Researchers have paid increasing attention to natural drugs in recent years because of their low cytotoxicity and high bioactivity. *Atractylodes macrocephala* (known as *baizhu* in China) is a widely used traditional Chinese medicine and it is reported to have various physiological and pharmacological effects. AT-II, the major lactone compound isolated from *Atractylodes macrocephala* exhibits remarkable anti-cancer effects in many types of tumors, but its role in endometrial cancer and the mechanisms underlying its role remain unclear [20]. In our study, we first verified that AT-II suppressed cell growth by reducing cell viability and colony formation capability in a time- and dose-independent manner. These findings suggest that AT-II could serve as a potential drug against endometrial cancer.

Cancer cells are characterized by increased glycolysis levels and lactate production even in conditions of adequate oxygen and this process is known as the Warburg effect [21,22]. The Warburg effect facilitates cancer cell malignancy by quickly promoting biosynthesis and energy production, thereby encouraging cell growth and increasing drug resistance [23]. Key glucose-metabolism-related enzymes, including PKM2 and LDHA, are commonly overexpressed due to enhanced levels of glycolysis. PKM2 is the last step-rate-limiting enzyme in the glycolytic process, which converts phosphoenolpyruvate to pyruvate. A previous study revealed that the high expression of PKM2 in endometrial cancer not only promoted metabolism reprogramming and metastasis, but also indicated poor prognosis [24]. LDHA catalyzed the conversion of pyruvate into lactate, maintaining an acidic microenvironment and contributing to cancer cell growth. Inhibition of LDHA activity effectively impairs the growth of tumors in various types of cancer cells [25]. In the present study, we found AT-II treatment suppressed glucose consumption, lactate, and ATP production. We also detected the mRNA and protein expression levels of these important enzymes and confirmed the downregulation of PKM2 and LDHA expression in response to treatment with AT-II. These results suggested that AT-II inhibited glycolysis by blocking the expression of PKM2 and LDHA.

Apoptosis is a complex biological process that occurs under physiological and pathological conditions. The progression and development of tumors are caused by tumor cells evading apoptosis. Therefore, inducing cell apoptosis becomes a critical strategy in cancer treatment [26]. The Bcl-2 family plays an important role in mitochondrional–mediated apoptosis by regulating the expression of anti-apoptotic (Bcl-2) and pro-apoptotic (Bax) members. The increased Bax and decreased Bcl-2 expression induced the activation of caspase-3, which promoted the cleavage of PARP, subsequently contributing to cell death [23,27]. Similar to previous studies, our results showed that AT-II induced cell apoptosis in a dose-dependent manner, accompanied by the decreased expression of Bcl-2, and the increased expression of Bax and cleaved-PARP. These data suggested that AT-II promoted apoptosis by regulating the mitochondrial apoptosis pathway.

The PADI family has five members: PADI1–4 and PADI6, but only PADI1–4 are active [28]. PADIs catalyze the conversion of protein-bound arginine residues to citrulline residues in proteins; this may have a big impact on the structure and function of the target protein [29]. Recently, increasing evidence has suggested that the crucial roles of PADIs are associated with the onset and progression of cancers. PADIs are highly expressed in a wide range of human malignant cancers, together with the fact that synthetic PADI inhibitors can lead to apoptosis and changes in mitochondrial structure in cancer cells; this strongly suggests that PADIs play important roles in tumorigenesis [30,31]. PADI3 seems to be cell-type dependent and is mostly expressed in solid tumors, such as those associated with bladder, pancreatic, cervical, head and neck, and clear-cell renal cancers, which are known to possess a hypoxic character [17], while it is weakly expressed in colon cancer, acting as a tumor suppressor gene [32,33]. In multiple cancer cell types, PADI3 modulates PKM2 citrulline to contribute to increased glycolysis and cell proliferation [18]. However, no previous studies have experimentally investigated the potential roles of PADI3 in endometrial cancer. In the present study, we determined that the expression of PADI3 mRNA was reduced in the AT-II treatment groups compared with that in the control groups using RNA-Seq analysis. Western blot and real-time PCR results were consistent with this result, highlighting that PADI3 may serve as an oncogene in endometrial cancer and in response to the anti-cancer activity of AT-II. Then, we analyzed how PADI3 expression was upregulated in patients with endometrial cancer, and that high expression of PADI3 was associated with poor prognosis, suggesting that PADI3 may be involved in regulating endometrial cancer progression. To confirm this hypothesis, we transfected endometrial cancer cells with si-PADI3 and found that PADI3 knockdown suppressed cell proliferation and glycolysis, and induced apoptosis. From these data, we verified that PADI3 played an important role in endometrial cancer tumorigenesis, serving as a novel target for endometrial cancer therapy. Moreover, via the rescue method, we further confirmed that PADI3 was the target of AT-II. Overexpression of PADI3 attenuated the effects of AT-II on the two cell lines, promoting their viability, enhancing glycolysis in these cells, and suppressing their apoptosis. 

The mitogen-activated protein kinase (MAPK) signaling network is the most important signaling pathway that contributes to multiple physiological and pathological behaviors; its aberrant activation is perhaps the major oncogenic driver of human cancers [34,35]. A previous study has shown that the PAD family member PADI2 played a critical role in the malignancy of endometrial cancer by activating the expression of p-ERK in endometrial cancer cells [36]. Therefore, we speculated whether PADI3 could play a similar role. Furthermore, in the present study, the MAPK signaling pathway was the most enriched in KEGG enrichment analysis. We also found that AT-II treatment and PADI3 knockdown remarkably decreased the phosphorylation level of ERK1/2. To assess whether ERK inactivation is involved in AT-II-induced glycolysis suppression and cell apoptosis, the ERK activator TBHQ was used. TBHQ and AT-II treatment significantly reversed the AT-II-induced inhibition of proliferation and glycolysis, increasing the expression of glycolytic enzymes PKM2 and decreasing the expression of cleaved-PARP. These data indicated that ERK blocking is essential for AT-II-induced glycolysis and apoptosis. 

In conclusion, AT-II exerts anti-cancer activity by blocking the PADI3-ERK signaling pathway. Thus, it may serve as a novel potential therapeutic target for endometrial cancer and targeting AT-II may represent a potential clinical strategy for treating patients with endometrial cancer.

## 4. Materials and Methods

### 4.1. Reagents and Antibodies

AT-II (purity ≥ 98%) was purchased from Chengdu Must Bio-technology Co., Ltd. (Chengdu, China). Tert-Butylhydroquinone (TBHQ) (purity ≥ 99.88%) was purchased from MCE (Monmouth Junction, NJ, USA). The stock solutions of AT-II (100 mM) and TBHQ (50 mM) were dissolved in dimethyl sulfoxide (DMSO). The following antibodies were used in this study: antibodies against PKM2 (15822-1-AP, Proteintech, Rosemont, IL, USA), LDHA (19987-1-AP, Proteintech), Bcl-2 (#15071S, CST), Bax (50599-2-Ig, Proteintech), cleaved-PARP (#5625P, CST), p-ERK (#4370S, CST), ERK (#4696S, CST), PADI3 (ab183209, Abcam, Cambridge, UK), and β-actin (AC038, Abclonal, Woburn, MA, USA).

### 4.2. Cell Culture

The two human endometrial cancer cell lines RL95-2 and AN3CA and the reagents associated with cell culture were obtained from Procell (Wuhan, China). RL95-2 cells were cultured in DMEM/F12 medium and AN3CA cells were cultured in MEM-modified medium. All cells were supplemented with 10% fetal bovine serum, 100 U/mL penicillin, and 100 µg/mL streptomycin. In addition, the culture medium for RL95-2 cells was supplemented with 5 µg/mL insulin. The cells were incubated at 37 °C in 95% humidified air and 5% CO_2_.

### 4.3. Cell Viability Assay

Cell Counting Kit-8 (CCK-8) (APE&BIO, Houston, TX, USA) was used to determine cell viability. Cells were seeded in 96-well plates at a density of 5 × 10^3^ cells/well and treated with different conditions. Subsequently, 10 µL of CCK-8 solution was added to each well and followed by incubation for 2 h at 37 °C. Then, the optical density value of cells was measured at 450 nm.

### 4.4. Colony Formation Assay

The RL95-2 and AN3CA cells were seeded in 6-well plates at a density of 500 cells/well. After incubation for 14 d, the cells were fixed with methanol for 10 min and stained with 1% crystal violet for 15 min. Then, the cells were washed with phosphate-buffered saline (PBS) and photographed. Colony numbers were counted using Image J (Version 1.37v).

### 4.5. Glycolysis Analysis

The RL95-2 and AN3CA cells were seeded into 6-well plates at a density of 3 × 10^5^ cells/well. The treated cells were collected and lysed. The lysis solution was centrifuged to remove the cell precipitate. According to the manufacturer’s instructions, the levels of glucose, lactate and ATP in the supernatant were measured using a glucose assay kit (Beyotime, Shanghai, China), lactate assay kit (Nanjing Jiancheng Bioengineering Institute, Nanjing, China), and ATP assay kit (Beyotime, Shanghai, China), respectively. The glucose, lactate, and ATP levels were calculated based on a standard curve and normalized relative to the protein concentrations.

### 4.6. Cell Apoptosis Assay

Cell apoptosis was detected using an Annexin V-FITC/PI apoptosis detection kit following the manufacturer’s instructions (Vazyme, Nanjing, China). Cells were collected, rinsed with PBS, re-suspended in 100 µL of binding buffer, and incubated with Annexin V-FITC and propidium iodide for 10 min at room temperature in the dark. Subsequently, 400 µL of binding buffer was added to each sample, and samples were tested within 1 h using FACS flow cytometer.

### 4.7. Western Blot

Total protein was extracted using RIPA buffer (Beyotime, Shanghai, China). Protein concentration was determined using the enhanced BCA protein assay kit (Beyotime, Shanghai, China). The proteins were separated via 10% SDS-PAGE gels and transferred onto polyvinylidene fluoride membranes (Millipore, Billerica, MA, USA). Then, the membranes were blocked in 1% bovine serum albumin for 1 h at room temperature and incubated overnight with primary antibodies against the target proteins at 4 °C, followed by incubation with horseradish peroxidase-conjugated secondary antibody for 1 h at room temperature. Then, the target proteins were examined using an ECL kit (Beyotime, Shanghai, China). β-actin was used as the loading control. The protein-relative expression levels were quantified by Clinx Gel Analysis software (Version 2.6.1.0).

### 4.8. RNA Sequencing

The RL95-2 cells were treated with 100 µM AT-II or DMSO for 48h. RNA was extracted using TRIzol reagent, and the samples were sent to Genechem (Shanghai, China) for RNA-seq. Three samples were supplied in each group. The differential expressed genes (DEGs) were defined based on the following criteria: |log2FoldChange| > 1 and *p* < 0.05.

### 4.9. Bioinformatics Analysis

The mRNA differential expression of PADI3 between paired or unpaired endometrial cancer tissues and normal tissues in the Cancer Genome Atlas (TCGA) dataset was visualized using Xiantao Academic online tool (https://www.xiantaozi.com/ (accessed on 20 February 2023)). According to the data from TCGA, the receiver operating characteristic (ROC) curve of PADI3 was detected to evaluate its clinical diagnostic efficiency. Survival rates were determined by the Kaplan–Meier method and survival times were compared using the Log-rank test. The correlations between PADI3 expression and clinicopathological features, and univariate and multivariate Cox regression analysis were examined to evaluate its prognostic value. The TIMER database (http://timer.cistrome.org/ (accessed on 19 February 2023)) was used to analyze the relationship of PADI3 expression with survival time.

### 4.10. Tissue Microarray and Immunohistochemistry Staining

Endometrial cancer tissue microarray (Cat HUteA045PG01) obtained from Shanghai Outdo Biotech Company was tested by immunohistochemistry staining. The study was approved by the Ethics Committee of Shanghai Outdo Biotech Company (Project identification code SHYJS-CP-1504010). The tissue microarray was deparaffinized and rehydrated, and antigen repair was performed under high pressure condition with EDTA pH 8.0 solution. Endogenous peroxidase was blocked with 3% H_2_O_2_. Next, the tissue microarray was incubated with the primary antibody of anti-human PADI3 (abcam Cat183209) at 4 °C overnight. After the incubation with horseradish peroxidase (HRP) conjugated secondary antibody at 37 °C for 30 min, the sections were counterstained by hematoxylin and eosin for subsequent observing and taking photos under microscope. 

### 4.11. Real-Time PCR

Total RNA was extracted from treated cells using TRIzol reagent (Vazyme, Nanjing, China). Real-time PCR was performed using a reverse transcription kit and SYBR qPCR master mix kit (Vazyme, Nanjing, China). β-actin was used as the loading control. Data expression was determined using the 2^−ΔΔCt^ method. The sequences of the primers used were as follows: PKM2 (NM_182471), forward, 5′-ATGTCGAAGCCCCATAGTGAA-3′, reverse, 5′-TGGGTGGTGAATCAATGTCCA-3′; LDHA (NM_001165415), forward, 5′-TTGACCTACGTGGCTTGGAAG-3′, reverse, 5′-GGTAACGGAATCGGGCTGAAT-3′; PADI3 (NM_016233), forward, 5′-CCCTCGTGGACATTTATGGGT-3′, reverse, 5′-ATCTCCAAAGTCGCGTCAAAG-3′; β-actin (NM_001101), forward, 5′-AGCGAGCATCCCCCAAAGTT-3′, reverse, 5′-GGGCACGAAGCTCATCATT-3′.

### 4.12. PADI3 siRNA and Overexpression Plasmid

The human PADI3 siRNA and negative control (NC) were purchased from JTSBIO (Wuhan, China). The sequences were as follows: si-PADI3^1#^: forward, 5′-CCACAAACUUGUCCUCCAUTT-3′, reverse, 5′-AUGGAGGACAAGUUUGUGGTT-3′; si-PADI3^2#^: forward, 5′-CCAGGUGCUCUCCAAUAAATT-3′, reverse, 5′-UUUAUUGGAGAGCACCUGGTT-3′; si-PADI3^3#^: forward, 5′-GCACCAAUGUGUGCAGAAATT-3′, reverse, 5′-UUUCUGCACACAUUGGUGCTT-3′; NC: forward, 5′-UUCUCCGAACGUGUCACGUTT-3′, reverse, 5′-ACGUGACACGUUCGGAGAATT-3′. The human PADI3 overexpression plasmid pCMV-PADI3-3FLAG and empty vector pCMV3-CON-3FLAG were purchased from Genechem (Shanghai, China). The cells were transfected using Trans-Mate (JTSBIO, Wuhan, China) following the manufacturer’s instructions.

### 4.13. Statistical Analysis

All experiments were performed at least three times, and the results are presented as the mean ± standard deviation. GraphPad Prism 8.0 was used for statistical analysis. One-way analysis of variance and Tukey’s test were used for multiple comparisons in several groups. An unpaired *t* test was employed between the two groups. *p* < 0.05 was considered as statistically significant.

## Data Availability

The data of RNA-seq has been deposited in GEO under accession number GSE252780.

## Supplementary Materials

The following supporting information can be downloaded at: https://www.mdpi.com/article/10.3390/molecules29050939/s1, Figure S1: The statistical results of colony formation assay are shown after AT-II treatment in RL95-2 cells and AN3CA cells. Figure S2: The correlation between PADI3 and the histologic grade was analyzed using the Xiantao Academic online tool. Figure S3: The efficiency of the three PADI3 siRNAs are shown and quantified by Clinx Gel Analysis software (Version 2.6.1.0) after transfecting PADI3 siRNAs in RL95-2 cells and AN3CA cells for 48 h. Figure S4: The statistical results of colony formation assay are shown after transfecting si-PADI3 in RL95-2 cells and AN3CA cells for 48 h. Figure S5: The protein-relative expression levels of PADI3 were detected by Western blot and quantified by Clinx Gel Analysis software (Version 2.6.1.0). Table S1: The correlation between the clinical features and the expression of PADI3.
