# Supplementary material for: Atractylenolide II Suppresses Glycolysis and Induces Apoptosis by Blocking the PADI3-ERK Signaling Pathway in Endometrial Cancer Cells
Source: Molecules. 2024 Feb 21;29(5):939. doi: 10.3390/molecules29050939 (PMC10934053; doi:10.3390/molecules29050939)
Supplement: Supplementary file 1 [file molecules-29-00939-s001.zip › molecules-2835535-supplementary.pdf]

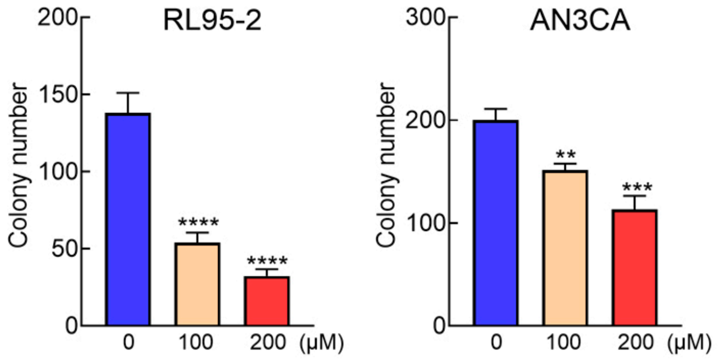

Figure S1. The statistical results of colony formation assay were showed after AT-II treatment in RL95-2 cells and AN3CA cells. All experiments were replicated independently at least three times and presented as mean ± SD (**$p < 0.01$, ***$p < 0.001$, ****$p < 0.0001$ vs 0 μM group).

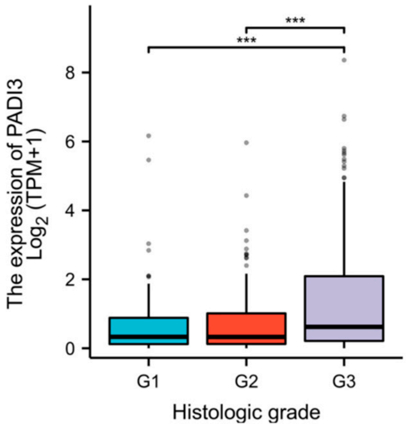

Figure S2. The correlation between PADI3 and the histologic grade was analyzed using Xiantao Academic online tool. (***p < 0.001 vs G1 or G2).

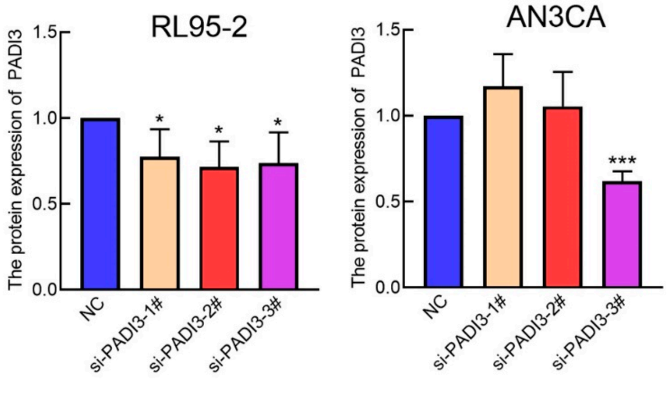

Figure S3. The efficiency of the three PADI3 siRNAs were showed and quantified by Clinx Gel Analysis software (Version 2.6.1.0) after transfecting PADI3 siRNAs in RL95-2 cells and AN3CA cells for 48 h. All experiments were replicated independently at least three times and presented as mean ± SD (*$p < 0.05$, ***$p < 0.0001$ vs NC group).

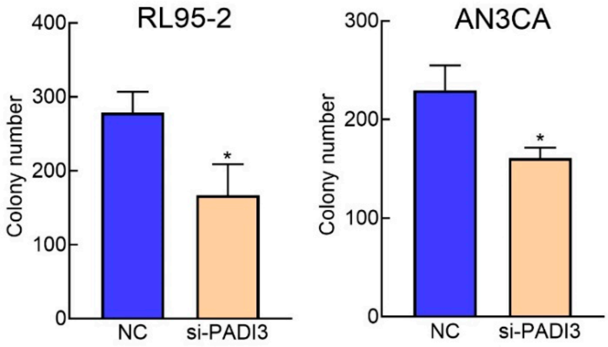

Figure S4. The statistical results of colony formation assay were showed after transfecting si-PADI3 in RL95-2 cells and AN3CA cells for 48 h. All experiments were replicated independently at least three times and presented as mean ± SD (*$p < 0.05$ vs NC group).

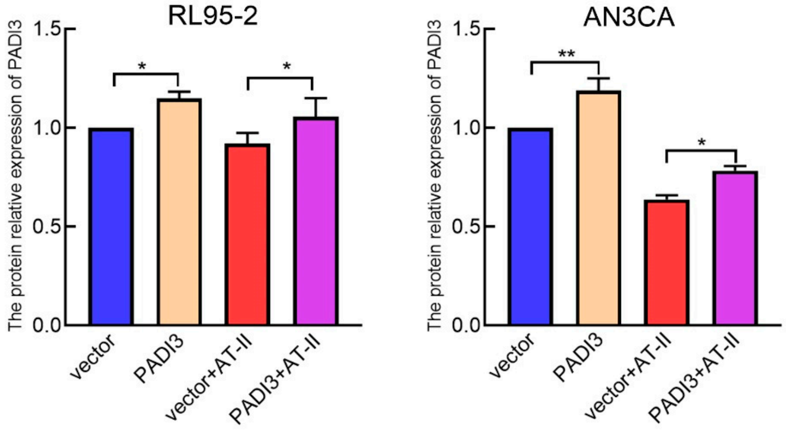

Figure S5. The protein relative expression levels of PADI3 were detected by Western blot and quantified by Clinx Gel Analysis software (Version 2.6.1.0). All experiments were replicated independently at least three times and presented as mean ± SD (*$p < 0.05$, **$p < 0.01$).

Table S1. The correlation between the clinical features and the expression of PADI3

Characteristics	Low expression of PADI3	High expression of PADI3	P value
n	277	277	
Age, n (%)			0.198
≤ 60	111 (20.1%)	96 (17.4%)	
> 60	165 (29.9%)	179 (32.5%)	
Clinical stage, n (%)			0.372
Stage I	181 (32.7%)	162 (29.2%)	
Stage II	25 (4.5%)	27 (4.9%)	
Stage III	57 (10.3%)	73 (13.2%)	
Stage IV	14 (2.5%)	15 (2.7%)	
Histologic grade, n (%)			**< 0.001**
G1	63 (11.6%)	36 (6.6%)	
G2	74 (13.6%)	47 (8.7%)	
G3	137 (25.2%)	186 (34.3%)	
